# Zinner Syndrome: Ipsilateral Renal Agenesis and Seminal Vesicle Cyst Presenting With Bony Metastasis

**DOI:** 10.7759/cureus.28949

**Published:** 2022-09-08

**Authors:** Shameer Deen, Ajay Arora, Rahul Lunawat

**Affiliations:** 1 Urology, Princess Royal University Hospital, London, GBR; 2 Radiodiagnosis, Kings College Hospital, London, GBR

**Keywords:** seminal vesicle cyst, mesonephric duct abnormality, zinner syndrome, ejaculatory duct obstruction, renal agenesis

## Abstract

Zinner syndrome is a rare congenital triad of mesonephric duct abnormality encompassing unilateral renal agenesis or dysgenesis, ipsilateral seminal vesicle cyst, and ejaculatory duct obstruction. Literature has reported 214 cases, with the most common presentation being lower urinary tract symptoms and abdominal pain. Most cases are incidentally diagnosed, and MRI has been the choice of radiological diagnosis. We report the case of an 81-year-old male who presented with a three-month history of a fungating elbow lesion, elbow pain, and weight loss. Imaging revealed an ipsilateral seminal vesicle cyst, absent kidney, and ejaculatory duct obstruction, i.e., Zinner syndrome with bone metastasis. A bone biopsy revealed a urothelial primary, and cyst aspiration and cytology revealed spermatozoa and malignant cells representing an adenocarcinoma. This patient was managed with symptom control, radiotherapy to the elbow, and palliative chemotherapy, but later succumbed to the condition.

## Introduction

Zinner syndrome is a rare congenital triad of mesonephric (Wolffian) duct abnormalities comprising unilateral renal agenesis or dysgenesis, ipsilateral seminal vesicle cyst, and ejaculatory duct obstruction. This was first described by Zinner himself in 1914 [1]. Since then, 214 cases have been incidentally diagnosed with a myriad of presentations ranging from asymptomatic or non-specific symptoms of fatigue, malaise, and abdominal pain, or masquerading as prostatitis with perianal pain or long-standing urinary tract symptoms such as dysuria, increased frequency and ejaculatory failure and infertility. At the far end of the spectrum is malignant change, where approximately 60 cases have been reported in the literature. We report the first case of Zinner Syndrome presenting as a fungating bone lesion.

## Case presentation

An 81-year-old man was admitted under the care of the orthopaedics team with a fungating elbow lesion and a 3-month history of left elbow pain, swelling, and weight loss. He also informed of a long-standing, constant dull ache in his elbow and no associated history of trauma to the elbow.

Physical examination revealed an unremarkable cardiorespiratory system. Musculoskeletal assessment of the left arm revealed poor active and passive movements of the elbow joint. The fungating elbow lesion had malodorous discharge with heaped edges and local inflammatory skin changes. Abdominal examination revealed a soft, non-tender abdomen and a right iliac fossa swelling. A bladder scan revealed minimal residual urine. DRE revealed an enlarged smooth prostate with no suspicious features.

On admission, blood tests demonstrated anaemia and elevated CRP likely on the background of malignancy and elbow wound (Table 1). Renal function, liver function, and PSA were within normal limits, and kappa and lambda chains did not identify myeloma as a cause.

**Table 1:** Blood Test results reveals anaemia, acceptable renal function, a marginally raised liver profile, normal PSA and a normal Kappa:Lambda Ratio thus ruling out a myeloma

Blood Test		Result	Normal values
Full Blood	Haemoglobin	94 g/L	133 – 167 g/L
	White blood cells	11.5 x 10^9^/L	3.7 – 9.5 x 10^9^/L
	Platelets	155 x 10^9^/L	140 – 400 x 10^9^/L
	Lymphocytes	0.90 x 10^9^/L	1 – 3.2 x 10^9^/L
	Neutrophils	4.50 x 10^9^/L	1.7 – 6.1 x 10^9^/L
Renal Profile	Creatinine	169 umol/L	61 – 123 umol/L
	Glomerular Filtration Rate	>90mL/min/1.73m^2^	mL/min/1.73m^2^
	Sodium	134 mmol/L	135 – 145 mmol/L
	Potassium	5.1 mmol/L	3.5 – 5.0 mmol/L
Liver Profile	Bilirubin	22 umol/L	0 - 20 umol/L
	Alkaline Phosphatase	278 U/L	30 – 130 U/L
	Alanine Amino Transferase	55 IU/L	5 – 55 IU/L
	Albumin	37 g/L	35 – 50 g/L
Specific Blood Tests	C Reactive Protein	265 mg/L	0 – 4 mg/L
	Prostate Specific Antigen	0.30 ug/L	0 – 5.5 ug/L
	Kappa Light Chains	16.30 mg/L	3.3 – 19.4 mg/L
	Lambda Light Chains	22.1 mg/L	5.7 – 26.3 mg/L
	Kappa:Lambda Ratio	0.737	0.26 – 1.65

The patient had a background of stroke, hypertension, high cholesterol, type II diabetes mellitus, and erectile dysfunction. He was an ex-smoker.

**Figure 1:**
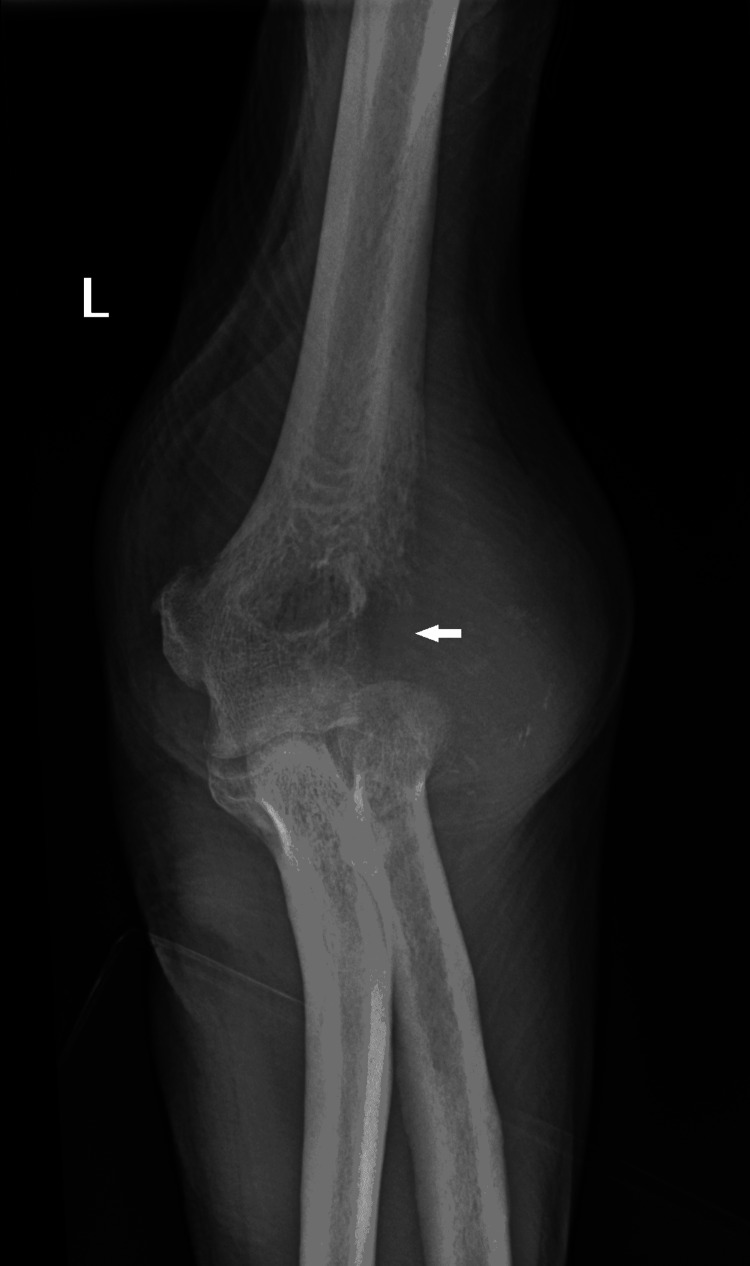
X-Ray Left Elbow (AP View) identifies an ill defined osteophytic lesion in the lateral epicondyle with loss of joint architecture and associated soft tissue swelling

The left elbow was imaged by X-Ray (Figure 1) which identified a bony lesion with infiltration into adjacent fascial and muscular planes. The differential diagnosis was a metastatic deposit from an unknown primary, Paget's disease of the bone, or a plasmacytoma.

**Figure 2:**
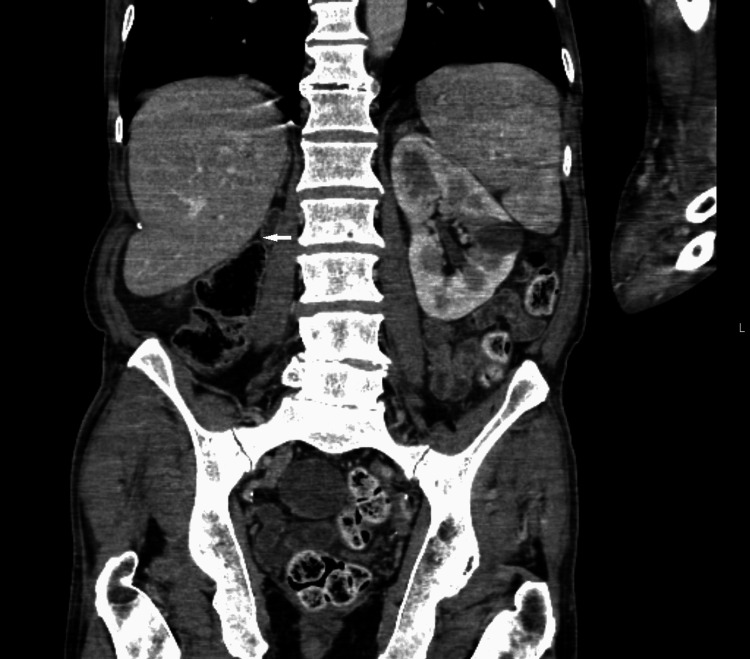
CT Abdomen (Coronal View): Absent right kidney, dilated seminal vesicle, and no locoregional lymphadenopathy

**Figure 3:**
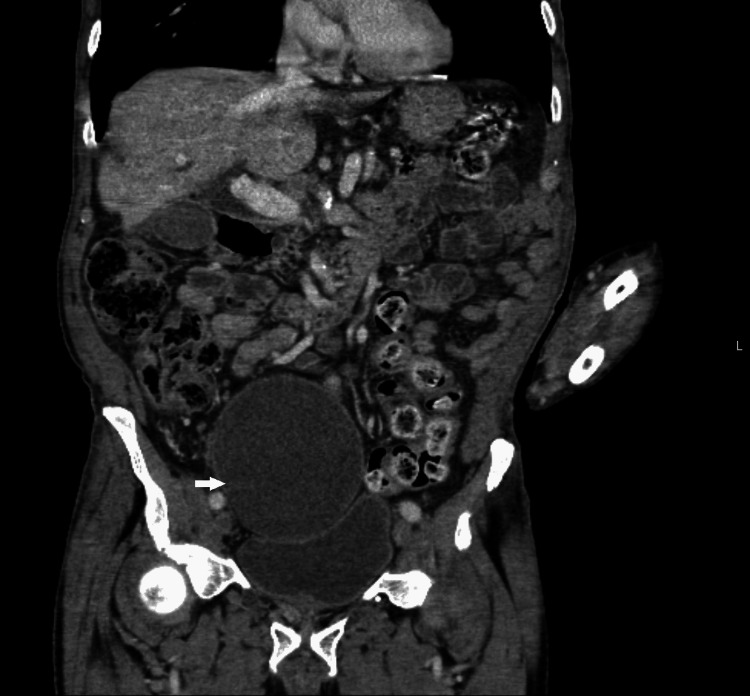
CT Abdomen (Coronal View): Large simple cyst originating from the right seminal vesicle and compressing the anterior bladder wall

**Figure 4:**
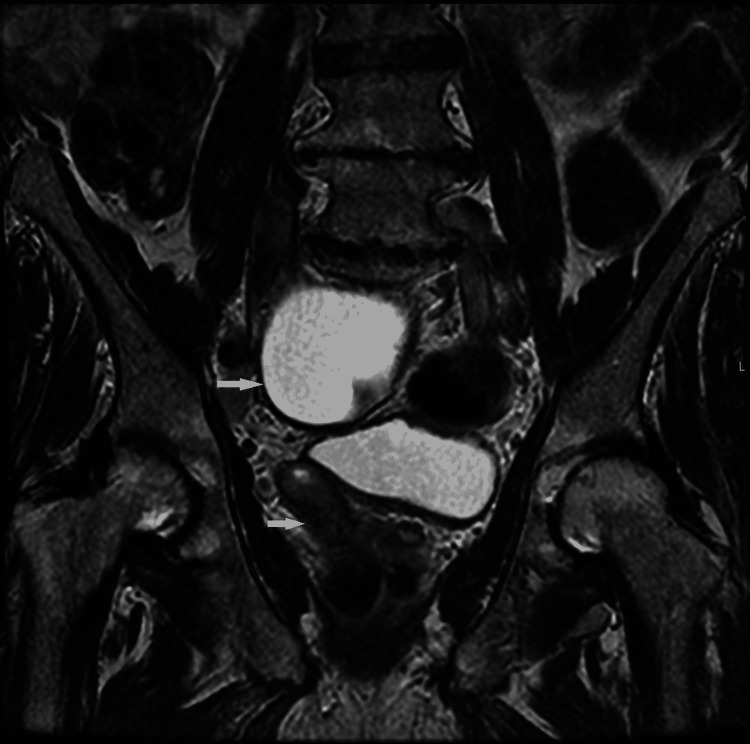
MRI Pelvis (Coronal View) confirming Zinner Syndrome Note that there is no local lymphadenopathy or bony metastasis

**Figure 5:**
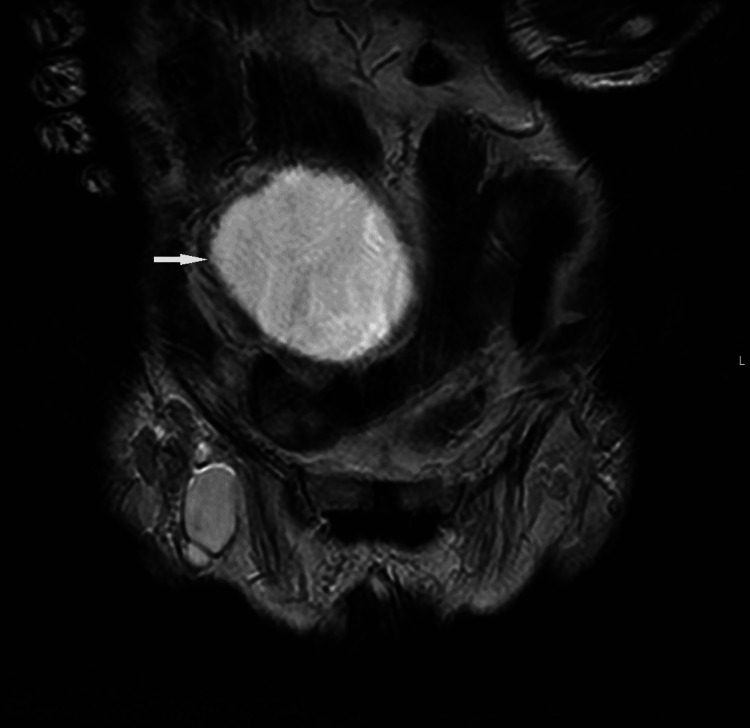
MRI Abdomen and Pelvis (Coronal View): Coronal view reveals a large simple seminal vesicle cyst with complex features

A contrast-enhanced CT of the chest, abdomen, and pelvis was performed to look for a primary malignancy. The CT demonstrated an absent right kidney (Figure 2) and large cystic lesion (Figure 3) measuring 9.7 cm x 9.2 cm x 17.2 cm arising from the right seminal vesicles and extending posteriorly and superiorly to the urinary bladder with ipsilateral renal agenesis. There was compensatory hypertrophy of the normally situated left kidney and simple renal and liver cysts. Furthermore, a single 1.5 cm enlarged left axillary lymph node and an intrapulmonary lymph node in the left lower lobe with associated peribronchial inflammation was identified. An MRI revealed a 9.0 cm T2 hyperintense cystic lesion in the pelvis indenting the urinary bladder (Figure 4). There was no evidence of solid components or septations within the cyst (Figure 5) and no note was made of local invasion or pathological enlargement of abdominal lymph nodes. The bladder was unremarkable.

The patient received analgesics, steroids, and intravenous antibiotics as part of his treatment and underwent biopsy of the bony lesion which identified it as urothelial primary malignancy. Lymph node biopsy was not appropriate as it was an 8mm node retained in a fatty hilum. Transperineal aspiration of cyst fluid identified spermatozoa and clusters of cuboidal or columnar cells with large, irregular, and hyperchromatic nuclei and vacuolated cytoplasm which was confirmatory for adenocarcinoma from the seminal vesicles. This confirmed the diagnosis of metastatic Zinner Syndrome.

This is the first reported case of Zinner Syndrome presenting with a fungating and metastatic bone mass. Surgical/laparoscopic excision or deroofing was not considered as owing to metastatic disease at presentation. The patient was treated with for symptom and pain control and received localized radiotherapy to the left elbow and palliative chemotherapy. The patient succumbed to the disease after 9 months of treatment.

## Discussion

A Medline(R) search with keywords ‘Seminal vesicle cyst and/or Renal Agenesis and/or Ejaculatory duct obstruction and/or Zinner syndrome and/or mesonephric duct abnormality.mp.’ identified 214 case reports of different pathology. This is the first reported case of fungating bone metastasis from a seminal vesicle in Zinner syndrome.

Zinner syndrome is the male counterpart of Mayer-Rokitansky-Küster-Hauser (MRKH) syndrome which is associated with ovarian and renal cell carcinoma [2]. Between the 4th and 13th gestational week, the collecting ducts of the kidney (metanephros) develop from the ureteric bud (an outgrowth of the mesonephric duct). The ureteric bud goes on to form the ureter, renal pelvis, calyces, and collecting tubules. The mesonephric duct gives rise to the epididymis, ductus deferens, and seminal vesicles. Agenesis and dysplasia are common, however incomplete migration of the ureteric bud results in improper differentiation of the metanephric blastema resulting in unilateral agenesis of all mesonephric duct structures. [3].

The incidence of Zinner syndrome is very low and has equal distribution in terms of site (right v/s left) [3]. Following Zinner, Sheikh et al. [4] published the results of their study where they undertook ultrasound screening of 280,000 children in Taipei with an aim to assess renal anomalies and detect associations of cystic dilatations in the pelvis with renal agenesis. Of these, only 13 cases were proved to have cystic dilatations in the pelvis with ipsilateral renal agenesis or dysplasia, and only 6 were seminal vesicle cysts. The frequency of this rare phenomenon in the study was 0.0046% (13/28,0000). However, only five cases of adenocarcinoma arising from the seminal vesicle cysts in patients with Zinner syndrome have been noted on a thorough review of the literature [3].

Kenney et al. [5] reported a large spectrum of image findings, ranging from cystic masses in the pelvis or thick and irregular walls or simple enlargement of the ipsilateral seminal vesicle. Other reports included a well-defined low-attenuation retrovesicular mass arising from seminal vesicles and cephalic to the prostate gland. On MRI, seminal vesicle cysts are identified with a variable signal intensity on T1- weighted images, and fluid signal intensity on T2- weighted images. Enhancement was not noted post IV gadolinium administration. Increased T1-weighted intensity is usually due to haemorrhage or proteinaceous content in the cyst [6]. MRI should be considered the gold standard in diagnosis as it can identify the connection between the ectopic ureter and seminal vesicle [7]. Differential diagnoses of these can be large prostatic cysts, congenital ureteroceles, Mullerian duct cysts, or Gartner’s duct cysts.

Eyal et al. [8] identified the various treatment modalities applied for surgical management of symptomatic seminal vesicle cysts and stated that primarily operative excision (preferably laparoscopic or robot-assisted) is superior. Open surgery is now a treatment of the past as it can be technically difficult owing to limited access to the retrovesical space. Furthermore, the robotic-assisted approach offers enhanced dexterity, precision, reduced blood loss, shorter stays, and manageable postoperative pain. Other treatments reported in the literature include aspiration (transrectal or transabdominal) and rarely transurethral deroofing, resection of the ejaculatory duct, or trans-seminal vesiculoscopic fenestration. All of these procedures were associated with recurrence.

## Conclusions

Urological developmental anomalies are diagnosed early in life and treated by a multi-disciplinary approach. Patients presenting to the hospital with unconstitutional symptoms are diagnosed by radiological modalities; hence the symbiotic relationship between radiodiagnosis and urological care. Zinner Syndrome is a rare congenital condition and delayed presentation is extremely rare, with malignant change noted on 11 occasions since it was officially described in 1914.

In this case of metastatic disease in an elderly gentleman, aspiration helped in confirming the diagnosis and treatment was catered after a multidisciplinary team review and best interest decisions were made. The patient was managed with symptom control, radiotherapy to the elbow, and palliative chemotherapy.
